# No Evidence That Circulating GLP-1 or PYY Are Associated with Increased Satiety during Low Energy Diet-Induced Weight Loss: Modelling Biomarkers of Appetite

**DOI:** 10.3390/nu15102399

**Published:** 2023-05-20

**Authors:** Jia Jiet Lim, Yutong Liu, Louise W. Lu, Ivana R. Sequeira, Sally D. Poppitt

**Affiliations:** 1 Human Nutrition Unit, School of Biological Sciences, University of Auckland, Auckland 1024, New Zealand; 2 Riddet Institute, Palmerston North 4442, New Zealand; 3 Department of Medicine, University of Auckland, Auckland 1010, New Zealand; 4 High-Value Nutrition National Science Challenge, Auckland 1010, New Zealand

**Keywords:** low energy diet, appetite, gastrointestinal peptide, biomarker, amino acid, visual analogue scale

## Abstract

Bariatric surgery and pharmacology treatments increase circulating glucagon-like peptide-1 (GLP-1) and peptide YY (PYY), in turn promoting satiety and body weight (BW) loss. However, the utility of GLP-1 and PYY in predicting appetite response during dietary interventions remains unsubstantiated. This study investigated whether the decrease in hunger observed following low energy diet (LED)-induced weight loss was associated with increased circulating ‘satiety peptides’, and/or associated changes in glucose, glucoregulatory peptides or amino acids (AAs). In total, 121 women with obesity underwent an 8-week LED intervention, of which 32 completed an appetite assessment via a preload challenge at both Week 0 and Week 8, and are reported here. Visual analogue scales (VAS) were administered to assess appetite-related responses, and blood samples were collected over 210 min post-preload. The area under the curve (AUC_0-210_), incremental AUC (iAUC_0-210_), and change from Week 0 to Week 8 (∆) were calculated. Multiple linear regression was used to test the association between VAS–appetite responses and blood biomarkers. Mean (±SEM) BW loss was 8.4 ± 0.5 kg (−8%). Unexpectedly, the decrease in ∆AUC_0-210_ hunger was best associated with decreased ∆AUC_0-210_ GLP-1, GIP, and valine (*p* < 0.05, all), and increased ∆AUC_0-210_ glycine and proline (*p* < 0.05, both). The majority of associations remained significant after adjusting for BW and fat-free mass loss. There was no evidence that changes in circulating GLP-1 or PYY were predictive of changes in appetite-related responses. The modelling suggested that other putative blood biomarkers of appetite, such as AAs, should be further investigated in future larger longitudinal dietary studies.

## 1. Introduction

Individuals with overweight and obesity are susceptible to cardiometabolic disorders, such as hypertension, dyslipidemia, and type 2 diabetes [1]. Dietary intervention for body weight (BW) loss and/or improved metabolic health is commonly the first line of treatment to delay the onset of these cardiometabolic disorders [2]. Low energy diets (LED) achieve rapid BW loss and a parallel improvement in multiple metabolic markers [3], with individuals able to successfully maintain BW loss long-term and likely to achieve the most favourable outcomes [4]. Regulation of appetite control may be key to this since successful BW loss has long been proposed to be associated with increased satiety [5,6]. All strategies to promote BW loss, including diet, exercise, pharmacological treatment and bariatric surgery, are accompanied by changes in appetite to varying degrees [7,8,9,10]. As hunger is a common obstacle to successful BW loss [11], interventions targeting appetite-regulating mechanisms to promote satiety during BW loss are of clinical importance.

BW loss changes body composition and, in turn, physiology, including circulating concentrations of glycemic-related parameters, gastrointestinal (GI) peptides, and amino acids (AAs) [12,13,14], several of which have been hypothesised as associated and/or causative of appetite change. GI peptides, such as glucagon-like peptide-1 (GLP-1) and peptide YY (PYY), have long been proposed to act as “satiety hormones” [15]. GLP-1 and PYY have convincingly been shown to promote feelings of satiety under specific conditions. For example, bariatric surgery which is highly successful for BW loss, results in a significant increase in circulating GLP-1 and PYY, and in turn promotes a parallel increase in satiety [16,17,18,19]. The pharmacological administration of (exogenous) GLP-1 analogues is also notable for promoting satiety [20,21,22,23].

Notably, however, neither surgical nor pharmacological treatments present the typical physiological conditions of an individual undertaking the widespread practice of an energy-restricted diet for BW loss. It is under these dietary conditions that the causal role of GLP-1 and PYY in promoting satiety has been questioned [20,24]. A widely adopted hypothesis purports a decrease in circulating GLP-1 and PYY during dietary-induced BW loss to be undesirable as it is likely associated with decreased satiety and increased energy intake (EI), potentially leading to BW re-gain [25,26]. However, our review [20] showed that a modest nutrient-induced postprandial increase in GLP-1 and PYY during dietary interventions rarely translated into a significant increase in satiety and/or decrease in *ad libitum* EI. Although dietary interventions designed to stimulate GLP-1 and PYY secretion have been proposed to promote satiety and BW loss [27,28,29], there has not yet been any evidence demonstrating that higher circulating concentrations of these (endogenous) peptides are indeed indicative of increased satiety, as per the more invasive bariatric and/or (exogenous) peptide treatments.

Additionally, the complicated physiological mechanism of appetite regulation may involve multiple biomarkers, such as circulating glucose and AAs [12,30,31,32]. Yet, the associations between these biomarkers and appetite have only been investigated under conditions of energy balance in acute postprandial studies. In these studies, a postprandial glucose “dip” below the pre-meal baseline was associated with increased self-reported hunger [32], and a postprandial AA “rise” was associated with increased self-reported satiety [30,31,33]. Despite no previous studies investigating these associations in the context of dietary-induced BW loss, a decrease in circulating glucose and multiple individual AAs was hypothesised to be associated with decreased satiety, in line with acute postprandial studies.

Given that we have previously reported a decrease in postprandial hunger when assessed as incremental change relative to fasting baseline in this 8-week LED weight loss study [12], the primary objective of the current analysis was to investigate whether the decrease in postprandial hunger may be associated with a concurrent increase in circulating GLP-1 and PYY, as previously reported in bariatric and pharmacological studies. This research investigated the association between circulating concentrations of GLP-1, PYY and satiety during diet-induced BW loss, for which a significant positive association has been purported but for which there is little underpinning data. Secondly, models were developed to test the associations between appetite response during BW loss and other blood biomarkers implicated in appetite regulation in postprandial studies including glucose, other glucoregulatory peptides and circulating AAs. To our knowledge, this study is novel in that it is the first study investigating the association of glucose, glucoregulatory peptides and AAs with appetite responses under the condition of diet-induced BW loss.

## 2. Materials and Methods

### 2.1. Trial Design

**Figure 1:**
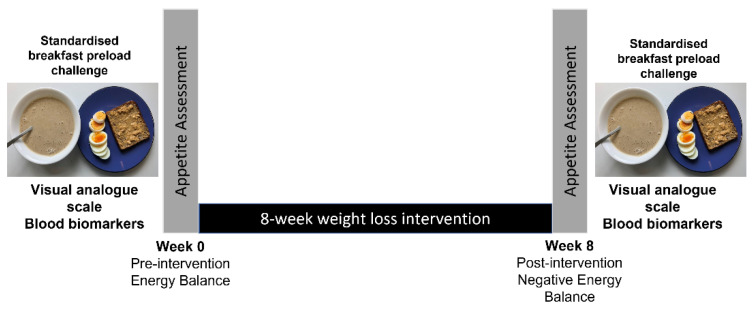
Study design. Participants completed a preload challenge at the pre-intervention (Week 0) and post-intervention (Week 8) phases of a low energy diet (LED) weight loss program.

This is a secondary analysis of a subset of individuals from a previously published LED intervention study [12]. Briefly, the main intervention was an unblinded, randomised, 4-arm, 8-week parallel trial aiming to investigate the effect of LED macronutrient composition on appetite response and BW loss in 121 women with obesity. Participants were randomly assigned in a 2 × 2 factorial design to either a higher protein (HP) or normal protein (NP) diet, in combination with lower carbohydrate (CHO) (LC) or normal CHO (NC). At the pre-intervention baseline (Week 0) and post-intervention (Week 8), all participants were required to attend the Human Nutrition Unit (HNU) clinic, Auckland, New Zealand for clinical assessments. The analysis presented in this study involved 42 participants who further undertook repeated postprandial blood sampling concurrent with appetite assessment using a Visual Analogue Scale (VAS) after consuming a standardised breakfast (Figure 1). Due to the smaller sample size in this secondary analysis, participants in all treatment groups were pooled and analysed as a single group.

The LED intervention complied with the Good Clinical Practice, received ethical approval from the New Zealand Human Disability Ethics Committee (Reference: 18/CEN/238) on 18 December 2018, and was prospectively registered with the Australia New Zealand Clinical Trial Registry (Reference: ACTRN12619000209190). Participants received a Participant Information Sheet and provided written informed consent before data collection.

### 2.2. Participant Recruitment and Eligibility

Recruitment methodology was previously published [12]. In summary, the LED intervention study included participants who were (i) female, (ii) aged 18–65 years, and (iii) had a body mass index (BMI) in the range 30–45 kg/m^2^ with a maximum BW of 130 kg. A single gender was selected for this LED intervention due to evidence that (i) appetite responses may differ between genders [34,35] and (ii) the metabolic response following LED intervention may also differ between genders [36]. Since females are more likely to participate in structured weight loss programs [37,38], women were enrolled in the intervention.

**Figure 2:**
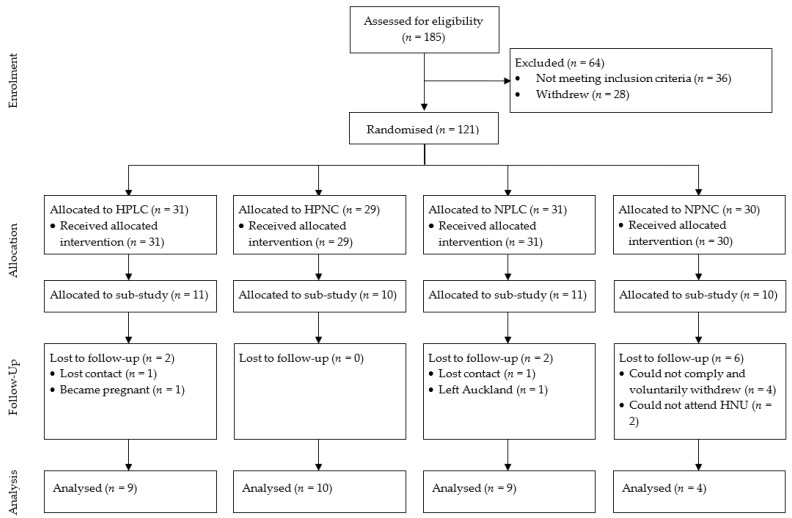
CONSORT flow diagram of participants. HPLC, higher protein lower carbohydrate; HPNC, higher protein normal carbohydrate; NPLC, normal protein lower carbohydrate; NPNC, normal protein normal carbohydrate; HNU, human nutrition unit.

Exclusion criteria were (i) BW change > 5% in the previous 3 months; (ii) current participation in an active diet program; (iii) current medications or conditions known to affect BW and/or appetite; (iv) prior bariatric surgery; (v) impaired liver or kidney function; (vi) significant current disease, such as stage 2 hypertension, type 2 diabetes, cardiovascular disease, cancer, or digestive disease; (vii) depression or anxiety; (viii) smokers or ex-smokers ≤ 6 months; (ix) pregnant or breastfeeding; (x) unable or unwilling to consume food items included in the study; or (xi) unwilling or unable to comply with other aspects of the study protocol. Additional requirements to participate in this sub-study were (i) consent to undergo venous cannulation; (ii) suitability to complete the cannulation procedure; (iii) no surgical or medical procedures of the digestive or endocrine system. The sub-study was offered to all eligible participants until the required sample size (*n* = 42) was achieved. Since the primary outcome of the study was to explore the association between the 8-week longitudinal change in VAS–appetite responses and circulating biomarkers, only participants who completed the preload challenge at both Week 0 and Week 8 are reported here (*n* = 32). The participant flow chart is shown in Figure 2.

### 2.3. LED Intervention

The LED was a partial diet replacement (PDR) regime, with breakfast comprising a participant-prepared oatmeal porridge supplemented with whey protein (Nutra Whey Natural, Nutratech Ltd., Tauranga, New Zealand), 2 commercial meal replacement sachets (Cambridge Weight Plan, New Zealand) as lunch and a mid-afternoon snack, and a dinner comprising a participant-prepared meal which was consumed *ad libitum* to appetite. Daily EI was approximately 40% of the calculated daily energy requirement following our previously published equation [12]:

40% of estimated daily energy requirement 
= 0.4 × Basal Metabolic Rate (BMR, Harris–Benedict Equation for women) × Estimated Physical Activity Level (PAL)
= 0.4 × (655 + (9.6 × weight in kg) + (1.8 × height in cm) − (4.7 × age in years)) × 4.184 (conversion from kcal to kJ) × 1.375 (assumed undertaking light activity at work)(1)

To promote compliance with the study protocol, participants attended fortnightly dietary consultation meetings with registered dietitians.

### 2.4. Clinic Visits and Preload Challenge Protocol

**Figure 3:**
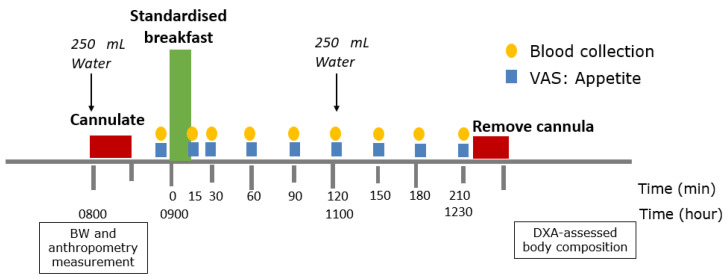
Summary of protocol during clinic visit at Week 0 and Week 8. BW, body weight; DXA, dual-energy X-ray absorptiometry; VAS, visual analogue scale.

A summary of the appetite assessment protocol at Week 0 and Week 8 is presented in Figure 3. Participants were requested to refrain from vigorous physical activity, alcohol, and unusually large or small meals 24 h prior to attending the clinic visit at Week 0 and Week 8, and adhere to the LED intervention until attending the clinic visit at Week 8. Participants arrived at HNU at 0800h after 10–14 h overnight fasting, consumed 250 mL water, had BW and anthropometry measurements conducted as previously reported [12], and had an indwelling venous cannula inserted. At 0900h, participants received a 1.8 MJ standardised mixed breakfast meal as a preload (27en% protein, 33en% fat, 37en% available CHO, and 3en% fibre), comprising toast with peanut butter, a hard-boiled egg, commercial meal replacement soup (chicken and mushroom) (Cambridge Weight Plan, Auckland, New Zealand) and 250 mL water. The energy content was similar to the median energy content of preloads as previously identified in our review of preload studies [20]. The higher protein content (27en%, 30 g) was intended to maximise the response of glucoregulatory peptides, GI peptides and AAs, and promote postprandial satiety. Participants consumed the breakfast in its entirety within 15 min. Subjective feelings of appetite were rated using paper-and-pen VAS and blood samples were collected in the fasted state (0855h, t = 0 min, baseline) and postprandial state (t = 15, 30, 60, 90, 120, 150, 180, 210 min), until 1230 h. The VAS was a 100 mm scale, with extreme feelings anchored at both ends of the scale, and consisted of questions previously used at the HNU to assess hunger, fullness, thoughts of food (TOF), and satisfaction [39]. The experiment setting adhered to international guidelines for appetite studies [40], also as previously described [39]. At Week 0 and Week 8, fat mass (FM) and fat-free mass (FFM) were assessed using dual-energy X-ray absorptiometry (DXA, iDXA software version 15, GE-Lunar, Madison, WI, USA) at the Auckland City Hospital, Grafton.

### 2.5. Laboratory Analysis

Blood samples were collected in a BD^TM^ Vacutainer (Becton, Dickinson and Company, Franklin Lakes, NJ, USA) containing fluoride oxidase for measuring plasma glucose, a BD^TM^ Vacutainer containing dipotassium ethylenediaminetetraacetic acid (K_2_EDTA) for measuring plasma AAs, and a BD^TM^ P800 Vacutainer containing a proprietary cocktail of peptide inhibitors for measuring plasma insulin, glucagon, gastric inhibitory polypeptide (GIP), total GLP-1 (GLP-1), and PYY. Plasma samples were obtained by centrifuging the Vacutainers at 1500× *g* for 10 min at 4 °C. Plasma and serum aliquots were stored at −80 °C until batch analysis. Plasma glucose was measured using a Cobas^®^ c311 analyser (Roche, Mannheim, Germany). Plasma insulin, glucagon, GIP, GLP-1 and PYY were measured using a MILLIPLEX^®^ MAP Human Metabolic Hormone Magnetic Bead Panel 96-Well Plate Assay (HMHEMAG-34K, Merck Millipore, Germany); the intra-assay and inter-assay coefficient of variant (CV) were ≤10.2% and ≤18.8%, respectively, in the laboratory. Plasma AAs were measured using an Ultra-High-Performance Liquid Chromatography assay with pre-column derivatisation using AccQ-Tag [41,42]; the intra-assay and inter-assay CV were ≤16.2% and ≤26.1%, respectively, in the laboratory.

### 2.6. Statistical Analysis

Descriptive data were reported as mean ± standard deviation (SD), and efficacy data as estimated marginal mean ± standard error of mean (SEM), unless otherwise stated. Continuous variables were checked for normal distribution and outliers. Extreme outliers were defined as data that lie > 3 interquartile range (IQR) away from the third quartile or the first quartile using a boxplot, and were excluded. The area under the curve (AUC_0-210_) and incremental area under the curve above fasted baseline (iAUC_0-210_) were also calculated for postprandial appetite ratings and postprandial concentrations of biomarkers using the trapezoid method. The difference in measurements between Week 0 and Week 8 was compared using paired T-tests, and the change at Week 8 from Week 0 was calculated as delta (∆). First, the associations between parameters of appetite ratings and biomarkers were explored using a correlation matrix. Then, multiple linear regression (MLR) models were developed to predict ∆AUC appetite from ∆AUC biomarkers after adjusting for multiple covariates. In Model 1, AUC_Week 0_ appetite and AUC_Week 0_ biomarkers were included as fixed effect covariates. We acknowledge that changes in BW and FFM are known to associate with changes in appetite and biomarkers [43,44,45]. Therefore, Model 2 included covariates in Model 1 plus age, BW_Week 0_, and ∆BW, and Model 3 included covariates from Model 1 plus age, FFM_Week 0_, and ∆FFM. The proportion of variance predicted by the ∆AUC biomarker after controlling for other covariates in the MLR model was expressed as partial *r^2^*, summarised using a heatmap. IBM’s Statistical Package for the Social Sciences (SPSS) software (version 28; IBM Corp., Armonk, NY, USA) [46] was used to perform statistical analyses. Statistical significance was set at *p* < 0.05. *A priori* sample size calculation using G*Power (Version 3.1.9.7, Kiel, Germany) [47] showed that to detect a partial *r^2^* = 0.2 with 80% power using a fixed effect MLR with 6 predictors, 34 participants were required. Accounting for dropouts, 42 participants were recruited for this analysis.

## 3. Results

### 3.1. Body Weight Change and Body Composition

Thirty-two participants with a mean (± SD) age of 40.0 ± 10.7 years and a mean BMI of 34.4 ± 3.2 kg/m^2^ completed the preload challenge protocol at both Week 0 and Week 8. BW and body composition at Week 0 and Week 8 are summarised in Table 1. The 8-week LED intervention significantly decreased mean (±SEM) BW (−8.4 ± 0.5 kg, *p* < 0.001), BMI (−3.2 ± 0.2 kg/m^2^, *p* < 0.001), total body FM (−6.6 ± 0.4 kg, *p* < 0.001), percentage FM (−3.7 ± 0.3%, *p* < 0.001), and total body FFM (−1.3 ± 0.2 kg, *p* < 0.001).

### 3.2. Effect of LED Intervention on VAS–Appetite Responses and Blood Biomarkers

#### 3.2.1. VAS

The effect of the 8-week LED intervention on VAS –appetite responses is summarised in Table 2. There was a non-significant trend towards an increase in fasted baseline hunger (∆ = 10 ± 6 mm, *p* = 0.100), and a significant increase in fasted baseline TOF (∆ = 8 ± 4 mm, *p* = 0.029). In contrast, postprandial hunger and TOF significantly decreased over 8 weeks when calculated as incremental change from the fasted baseline (iAUC_0-210_ hunger, ∆ = −2757 ± 1084 mm×min, *p* = 0.016; iAUC_0-210_ TOF, ∆ = −2323 ± 605 mm×min, *p* = 0.016), likely driven by the increase in fasted ratings from Week 0 to Week 8. Consequently, the direction of the effect on fasted hunger and TOF was opposite to the effect on postprandial iAUC_0-210_. There was no significant change in postprandial hunger or TOF when calculated as AUC_0-210_ (*p* > 0.05, both). There was no significant difference in any measures of fullness and satisfaction following the LED intervention (*p* > 0.05, all). The repeated measures plots are available in Supplementary Figure S1. 

#### 3.2.2. Blood Biomarkers

The effect of the LED intervention on GI peptides, glucose, glucoregulatory peptides and AAs is summarised in Table 3. When fasting, the LED intervention significantly decreased plasma glucose over 8 weeks (*p* = 0.006), in addition to AAs threonine, tryptophan, glutamic acid, alanine, tyrosine, and proline (all, *p* < 0.05), and significantly increased plasma serine (*p* = 0.006). When the postprandial response was calculated as an incremental change from the fasted baseline (iAUC_0-210_), the LED intervention significantly decreased plasma serine (*p* = 0.028), and significantly increased plasma glucose, GIP, and proline (*p* < 0.05, all). Conversely, when the response was calculated as AUC_0-210_, the LED intervention significantly decreased plasma phenylalanine, threonine, tryptophan, glutamic acid, alanine, tyrosine, proline, and citrulline, and significantly increased plasma GIP and serine (*p* < 0.05, all). The direction of change in AUC_0-210_ predominantly resembled the change in the fasted concentration for these parameters. The repeated measures plots are available in Supplementary Figures S2 and S3.

### 3.3. Associations between VAS–Appetite Responses and Blood Biomarkers

When the postprandial response was calculated as an incremental change from the fasted baseline, there was a significant decrease in iAUC_0-210_ hunger and TOF in response to the standardised breakfast meal, yet only 4 biomarkers were significantly altered at the same time. Conversely, when calculated as absolute change, there was no significant change in AUC_0-210_ VAS–appetite responses, yet as many as 10 biomarkers were significantly altered at the same time. Consequently, there was no obvious link between appetite-related response and biomarkers at the group level.

#### 3.3.1. Incremental Increase in Postprandial VAS–Appetite Responses Were Strongly Inversely Predicted by Fasted Responses

In the exploratory correlation analysis, iAUC_0-210_ hunger, fullness, TOF, and satisfaction had strong inverse correlations with their respective fasted ratings at both Week 0 and Week 8, as well as with change from Week 0 to Week 8 (∆fasted). The correlation between ∆fasted and ∆iAUC_0-210_ appetite-related responses was *r* = −0.83 for hunger (*p* < 0.001), *r* = −0.60 for fullness (*p* < 0.001), *r* = −0.64 for TOF (*p* < 0.001), and *r* = −0.69 for satisfaction (*p* < 0.001), confirming that the postprandial decrease in iAUC_0-210_ hunger and TOF previously observed was indeed strongly related to the increase in fasted baseline hunger and TOF. This inverse correlation was unexpected and likely unusual for physiological parameters. 

**Figure 4:**
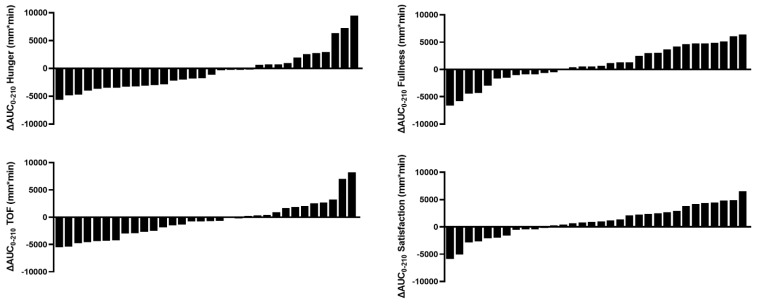
Individual participant’s ∆AUC_0-210_ appetite-related responses to the 8-week LED intervention. ∆AUC_0-210_, change in area under the curve measured over 210 min from Week 0 to Week 8.

Therefore, to investigate whether the LED-driven decrease in hunger or TOF was positively associated with an increase in circulating GLP-1 and PYY, ∆AUC_0-210_ data were used in the MLR where postprandial response was not inversely driven by fasting levels. Similarly, ∆AUC_0-210_ data were used to explore the association between VAS–appetite responses and other biomarkers. Although the LED did not significantly change mean ∆AUC_0-210_ appetite-related responses, there was wide between-individual variability with approximately half of the cohort reporting an increase, and the other half a decrease, in postprandial feelings of hunger, fullness, TOF, and satisfaction (Figure 4). When biomarkers were calculated as ∆AUC_0-210_, an outlier in the GLP-1 and glycine data was identified. These outliers were removed from the subsequent modelling analyses to maintain normally distributed data.

#### 3.3.2. High Circulating GLP-1 was Unexpectedly Positively Associated with Hunger and TOF and Inversely Associated with Fullness and Satisfaction

**Figure 5:**
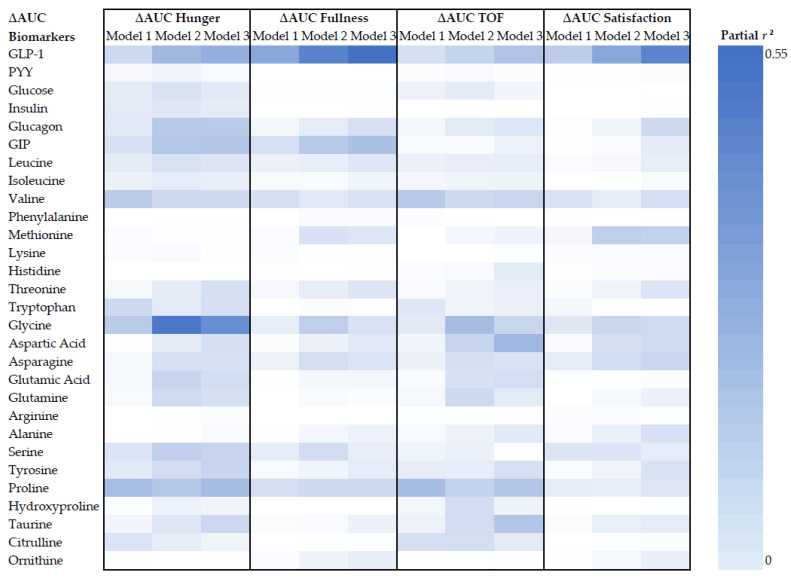
Heat map. Proportion of variance in appetite explained by biomarkers (∆AUC) was presented as partial *r^2^*, following multiple linear regression models. Model 1: ∆AUC Appetite = Week 0 AUC Appetite + Week 0 AUC Biomarker + ∆AUC Biomarker; Model 2: ∆AUC Appetite = Age + Baseline BW + ∆BW + Week 0 AUC Appetite + Week 0 AUC Biomarker + ∆AUC Biomarker; Model 3: ∆AUC Appetite = Age + Baseline FFM + ∆FFM + Week 0 AUC Appetite + Week 0 AUC Biomarker + ∆AUC Biomarker. ∆AUC, change in area under the curve from Week 0 to Week 8; GLP-1, glucagon-like peptide-1; PYY, peptide YY; GIP, gastric inhibitory polypeptide.

The proportion of variance in ∆AUC_0-210_ appetite-related responses explained by ∆AUC_0-210_ biomarkers is summarised as partial *r*^2^ using a heatmap (Figure 5). GLP-1, GIP, valine, glycine and proline were among the biomarkers consistently and significantly associated with two or more VAS–appetite responses, after adjusting for covariates. Contrary to the hypothesis, ∆AUC_0-210_ GLP-1 was positively associated with ∆AUC_0-210_ hunger and TOF, while inversely associated with ∆AUC_0-210_ fullness and satisfaction in all models (*p* < 0.05, all) (Table 4). This was an unexpected finding. In Model 1, the association between ∆AUC_0-210_ GLP-1 and ∆AUC_0-210_ appetite-related responses yielded a partial *r^2^* between 0.15 and 0.28. The association was stronger after adjusting for BW in Model 2 (partial *r^2^* = 0.28–0.43) and after adjusting for FFM in Model 3 (partial *r*^2^ = 0.33–0.51). Similarly yet unexpectedly, ∆AUC_0-210_ GIP was also positively associated with ∆AUC_0-210_ hunger and inversely associated with ∆AUC_0-210_ fullness in Models 2 and 3 (*p* < 0.05, all) (Table 4). Furthermore, branched-chain amino acid (BCAA) valine was also positively associated with ∆AUC_0-210_ hunger and TOF in all models (*p* < 0.05, all) (Table 4). The strength of association was smaller for GIP and valine than for GLP-1. PYY was not significantly associated with any VAS–appetite response in any of the predictive models (*p* > 0.05, all) (Table 4).

#### 3.3.3. Glycine as a Potential Biomarker for VAS–Appetite Responses

Glycine, a non-essential amino acid (NEAA), was identified as a biomarker with a high partial *r*^2^ value and inversely associated with hunger (Table 4). In Model 1, ∆AUC_0-210_ glycine was inversely correlated with ∆AUC_0-210_ hunger (partial *r*^2^ = 0.20, *p* = 0.014), but not associated with other appetite-related responses. The association between ∆AUC_0-210_ glycine and ∆AUC_0-210_ appetite-related responses was stronger after adjusting for BW in Model 2, whereby ∆AUC_0-210_ glycine was inversely associated with ∆AUC_0-210_ hunger (partial *r*^2^ = 0.53, *p* < 0.001) and TOF (partial *r*^2^ = 0.26, *p* = 0.007), while positively associated with ∆AUC_0-210_ fullness (partial *r*^2^ = 0.19, *p* = 0.027) and satisfaction (partial *r*^2^ = 0.16, *p* = 0.042). After adjusting for FFM in Model 3, ∆AUC_0-210_ glycine remained inversely associated with ∆AUC_0-210_ hunger (partial *r*^2^ = 0.45, *p* < 0.001) and TOF (partial *r*^2^ = 0.16, *p* = 0.043), while its positive association with satisfaction was trending towards significance (partial *r*^2^ = 0.15, *p* = 0.055). Another NEAA, ∆AUC_0-210_ proline, was also inversely associated with ∆AUC_0-210_ hunger and TOF and positively associated with ∆AUC_0-210_ fullness in all models (*p* < 0.05, all).

## 4. Discussion

The biomarkers of appetite in the current analysis were modelled using our prior 8-week LED weight loss study [12] since the intervention led to a clinically significant decrease of 8.2 kg BW (8% of baseline BW), paired with a favourable decrease in self-reported hunger when assessed as a postprandial change from fasted baseline levels. 

Surprisingly, this decrease was largely predicted by the inverse increase in self-reported fasted baseline hunger. Therefore, VAS–appetite responses were modelled using absolute (AUC_0-210_) and not incremental (iAUC_0-210_) measures, which, in turn, allowed the fasted VAS–appetite parameters to vary as a response to the 8-week LED intervention rather than fixing the baseline values to zero. Despite no significant change in mean postprandial (AUC_0-210_) VAS–appetite responses after LED intervention, approximately half of the population reported an increase in hunger and half reported a decrease. This between-individual variability was likely of great physiological relevance. For example, the lowest and highest change in mean postprandial (AUC_0-210_) hunger was −5655 mm×min and +9473 mm×min over 210 min, translated to an average of −27 mm and +45 mm difference in VAS-assessed hunger, while notably only a 10 mm difference was hypothesised to be clinically significant for changing eating behaviour [40]. Consequently, this analysis was deemed necessary to understand the physiological factors that drove this huge between-individual variability, in agreement with Gibbons et al. [48]. 

Whilst it has been commonly hypothesised that a decrease in self-reported hunger is inversely associated with an increase in circulating GLP-1, the current study showed that GLP-1 and PYY did not predict the hypothesised change in VAS–appetite responses as previously reported in bariatric surgery [17,18,49,50,51] or pharmacological [21,22,23] treatment studies. The current study also showed, using MLR modelling, that a decrease in both GIP and the BCAA valine, and an increase in NEAAs glycine and proline, constituted the biomarker profiles best associated with decreased hunger at the end of the 8-week LED weight loss intervention.

### 4.1. The Utility of Measuring Circulating GLP-1 and PYY during Appetite Assessments

In this study, the mean postprandial concentrations of GLP-1 and PYY did not differ before or after the 8-week LED intervention and BW loss, as also shown in previous LED studies [52,53,54,55]. Puzzlingly, the current study found that GLP-1 was positively correlated with self-reported hunger. This observation was unexpected, given the biological mechanisms by which GLP-1 interacts with GLP-1 receptors to promote satiety [56,57,58]. Notably, changes in BW and FFM are potential confounders for VAS–appetite responses [45]. When adjusting for BW and FFM loss, the positive association between GLP-1 and self-reported hunger and TOF became stronger. The partial *r*^2^ (0.28–0.43 in Model 2; 0.33–0.51 in Model 3) suggested that this association was unlikely to be a false positive outcome. 

This unexpected observation contradicted the bariatric Roux-en-Y gastric bypass (RYGB) studies, whereby higher postprandial concentrations of GLP-1 and PYY were inversely associated with self-reported hunger or positively associated with self-reported fullness [17,49,50]. Furthermore, Papamargaritis and le Roux [59] recently showed that hunger suppression following RYGB was attenuated by GLP-1 and PYY receptor antagonists, supporting the hypothesis that GLP-1 and PYY are involved in suppressing hunger or promoting satiety after RYGB. Yet, similar supporting evidence is missing in dietary-induced BW loss studies, whereby these studies reported no significant association between GI peptides and VAS–appetite responses [58,59,60,61,62]. Nevertheless, the positive association between GLP-1 and self-reported hunger in the current study was unexpected. 

GLP-1 and PYY have been described to promote satiety via the endocrine pathway and the neural pathway. In the endocrine pathway, GLP-1 and PYY released from the enteroendocrine L-cells enter the peripheral circulatory system, and diffuse across the blood–brain barrier to target the arcuate nucleus in the hypothalamus [57,58]. In the neural pathway, GLP-1 and PYY activate the local vagus nerve proximate to their site of secretions, relaying neural signals to the nucleus of the solitary tract in the brainstem to promote satiety [57,58]. In the current study, total GLP-1 was measured, comprising both the active and inactive forms following degradation by dipeptidyl peptidase-4 (DPP-IV) in peripheral circulation. Total GLP-1 was assumed to have captured both GLP-1 currently active in circulation and GLP-1 which had interacted with the neural pathway but subsequently degraded [63]. Notably, there are huge practical challenges in measuring the flux and utilisation of GLP-1 and PYY around their site of secretion and at the peptide receptors. Whilst we do not reject the role of these biological mechanisms in promoting satiety, the significance of the current findings was that simply measuring the peripheral circulating concentrations of GLP-1 and PYY, as has been undertaken in many appetite studies following dietary intervention, could not reliably predict the change in VAS–appetite responses.

### 4.2. The Utility of Measuring Circulating GIP during Appetite Assessments

The current study also showed that postprandial concentrations of GIP increased following the 8-week LED intervention and BW loss, in agreement with the Danish study of Iepsen et al. [64] in a cohort of 20 healthy individuals with obesity. There are very limited dietary-induced BW loss studies investigating the effect of GIP and its association with appetite. GIP is closely associated with GLP-1; both are incretin hormones known to trigger insulin secretion from pancreatic β-cells and to affect gastric motility, potentially influencing appetite perception [65,66]. Activation of the GIP receptor in the hypothalamus has been shown to reduce food intake in mouse models [67]. Moreover, GIP and GLP-1 dual agonists are successful pharmacology therapies for type 2 diabetes treatment, employed to promote BW loss [68]. In the current study, an increase in postprandial GIP was observed, but along with GLP-1, it was surprisingly also positively associated with self-reported hunger. The mechanism is unknown. 

### 4.3. The Utility of Measuring Circulating AAs during Appetite Assessments

Dietary and circulating AAs have long been implicated in appetite regulation in the postprandial phase [30,31,33]. Whilst diet-derived AAs can stimulate the secretion of GLP-1 to promote satiety [69], the brain AA sensing mechanism is also involved in appetite regulation. A higher concentration of leucine in the brain has been shown to inhibit food intake in rodent models [70]. A higher concentration of tryptophan is also hypothesised to increase brain serotonin, a neurotransmitter known to suppress appetite [71], and the deficiency of essential AAs, hence, relatively higher levels of non-essential AAs, in a meal lead to premature termination of the present meal [72].

However, the role of AAs in long-term appetite regulation following dietary-induced BW loss has not previously been assessed, nor have its mechanisms of action. The decrease in the fasted concentration of many circulating AAs generally reflects an improvement in cardiometabolic risk [13,73,74,75,76]. In our current study, when the postprandial concentration of AAs was assessed as AUC, the LED intervention significantly decreased plasma phenylalanine, threonine, tryptophan, glutamic acid, alanine, tyrosine, proline, and citrulline, and significantly increased plasma GIP and serine. Yet, MLR showed that the strength of association between most AAs and VAS–appetite responses was not significant, except for valine, glycine and proline. Glycine had the strongest association with VAS–appetite responses, whereby glycine was inversely associated with self-reported hunger, which was also previously reported in recent postprandial studies [33,77]. Karnani et al. [78] hypothesised that glycine may lower reward-seeking behaviour by reacting with the hypocretin/orexin neurons in the hypothalamus. Furthermore, low concentrations of circulating glycine may also be an important marker of obesity and type 2 diabetes [79,80]. Therefore, dietary interventions that can promote an increase in circulating glycine may be interesting to explore with a possible role in promoting satiety, in turn BW loss and decreasing type 2 diabetes risk. There was no prior evidence supporting the role of valine and proline on appetite responses; hence, future investigations may be required to confirm this association.

### 4.4. Considerations When Modelling the Biomarkers of Appetite

During the exploratory correlational analysis, the strong inverse association between fasted and postprandial VAS–appetite responses when calculated as a change from a fixed baseline (iAUC_0-210_) was surprising. Interestingly, this relationship was previously reported by King et al. [81] in a trial which investigated the effect of exercise-induced BW loss on appetite control in 58 individuals with obesity. The authors concluded that exercise-induced BW loss promoted an orexigenic drive to eat, but also increased meal-induced satiety. We were cautious as a result of this and did not conduct the MLR models based on iAUC_0-210_ values. 

An important difference between biomarkers and VAS assessments is the observation that VAS are limited by a finite scale (0–100 mm), whereas biomarkers are not. Therefore, when the initial hunger rating is low, the finite VAS scale cannot detect a substantial suppression in postprandial hunger relative to the fasted baseline prior to meal ingestion, in line with observations by Dalton et al. [82] and Barkeling et al. [83]. Whether this observation is physiologically relevant or is a methodological limitation is unclear.

We propose that studies which report iAUC VAS–appetite must clearly also describe the ‘raw’ fasted appetite ratings and consider whether the change in iAUC VAS–appetite is physiologically correct or rather a limitation of the methodology linked to the difference in appetite ratings in the fasted state. Although iAUC has been widely used for many biomarkers, the international methodology for appetite studies has favoured the use of AUC over iAUC when reporting VAS [40]. We acknowledge that the outcomes of MLR models may have been different had incremental postprandial changes from a fixed fasting baseline (iAUC values) been used. 

### 4.5. Strengths and Limitations

The current study had several strengths. Despite reporting a subset of participants from our 8-week LED weight loss study, anthropometry and appetite outcomes were comparable with the full cohort. Of note, the increase in fasting hunger was statistically significant in the full cohort of 121 women but was not significant in this smaller subset (*p* = 0.010) due to the large variability. Furthermore, MLR was performed to evaluate the 8-week longitudinal association between VAS–appetite and an array of blood biomarkers. Whilst a limited number of studies had previously investigated the association between VAS–appetite and GI peptides following dietary weight loss interventions [58,59,60,84], the current study is the first to explore the association between glucose, glucoregulatory peptides, AAs and VAS using an LED-induced weight loss model. 

Conversely, the study is limited to a single-gender intervention, and only women with obesity undergoing a specific LED regime were evaluated. Hence, the current findings may not be generalisable to a wider population undergoing varied dietary BW loss regimes. Furthermore, with limited sample size and an unbalanced number of completers in this sub-study cohort, the statistical power to assess the effect of the intervention on the postprandial response of biomarkers was limited. We also acknowledge that LC, but not HP, significantly promoted postprandial TOF and satisfaction in the full LED cohort of this study [12]. However, pooling the intervention groups in this smaller cohort is not expected to alter the main conclusion of the current analysis. Considering that GLP-1 and PYY were hypothesised to be predictive of subjective feelings of appetite, GLP-1 and PYY would have been inversely associated with hunger or positively associated with fullness, irrespective of macronutrient composition of the diet.

### 4.6. Recommendations for Future Studies

Mixed-gender, large sample size, long-term longitudinal studies are needed to better evaluate the utility of purported ‘satiety peptides’ and other biomarkers, such as AAs, in predicting VAS–appetite responses. Furthermore, assessment of appetite and biomarker data at multiple visits (for example, at Week 0, Week 4 and Week 8) would improve the robustness of the regression model. Other putative anorexigenic (e.g., β-hydroxybutyrate, and leptin) and orexigenic (e.g., active ghrelin) appetite biomarkers could also be measured and added to the multi-metabolite model. In addition, future methodological studies could investigate whether the inverse relationship between fasted and postprandial changes in VAS–appetite responses from a fixed fasted baseline is of physiological relevance or is a methodological limitation.

## 5. Conclusions

In conclusion, there was no evidence that circulating concentrations of GLP-1 and PYY were associated with enhanced satiety following an 8-week LED intervention which induced 8% BW loss in a cohort of women with obesity. This study, along with our previous findings, has shown changes in circulating concentrations of GLP-1 and PYY as commonly reported in dietary appetite studies are not reliable predictive markers of VAS–appetite responses. Notably, the flux and utilisation of GLP-1 and PYY at peptide secretion and receptor sites may be more relevant to their contribution as predictors of subjective feelings of appetite and eating behaviour than commonly measured circulating concentrations. The current study identified that a decrease in GLP-1, GIP and BCAA valine, in addition to increased NEAAs glycine and proline, constituted the biomarker profile predictive of increased postprandial satiety specific to this LED intervention. Despite GLP-1 and PYY being routinely measured and termed as “satiety peptides” in dietary studies, there is as yet little evidence to demonstrate the association between circulating concentrations of these peptides and subjective feelings of appetite in these interventions. The current modelling of other blood biomarkers including AAs is novel and provides data to underpin the design of future studies to further investigate putative biomarkers of appetite regulation under the conditions of dietary-induced weight loss.

## Figures and Tables

**Table 1:** Body weight and composition at Week 0 and Week 8.

Characteristics	Week 0	Week 8	∆Week 8	*p*-Value
Weight (kg)	90.9 ± 8.7	82.6 ± 8.9	−8.4 ± 0.5	<0.001
BMI (kg/m^2^)	34.4 ± 3.2	31.2 ± 3.4	−3.2 ± 0.2	<0.001
FM (kg)	41.6 ± 6.3	35.0 ± 6.5	−6.6 ± 0.4	<0.001
Percentage FM (%)	45.9 ± 3.9	42.2 ± 4.9	−3.7 ± 0.3	<0.001
FFM (kg)	48.7 ± 4.8	47.3 ± 4.8	−1.3 ± 0.2	<0.001

BW and body composition at Week 0 and Week 8 are reported as mean ± SD (*n* = 32). The changes from baseline Week 0 to Week 8 (∆Week 8) are reported as mean ± SEM, analysed using paired T-test. BMI, body mass index; FM, fat mass; FFM, fat-free mass.

**Table 2:** Participant appetite responses at Week 0 and Week 8.

VAS–Appetite Responses	Week 0	Week 8	∆Week 8	*p*-Value
Fasted hunger (mm)	41 ± 19	51 ± 27	10 ± 6	0.100
AUC_0-210_ hunger (mm×min)	6213 ± 3207	5647 ± 3215	−566 ± 633	0.378
iAUC_0-210_ hunger (mm×min)	−2403 ± 3949	−5160 ± 5644	−2757 ± 1084	0.016
Fasted fullness (mm)	33 ± 17	31 ± 20	−2 ± 3	0.548
AUC_0-210_ fullness (mm×min)	12,970 ± 3525	13,824 ± 3900	854 ± 608	0.170
iAUC_0-210_ fullness (mm×min)	6146 ± 4181	7449 ± 4905	1303 ± 663	0.058
Fasted TOF (mm)	52 ± 16	60 ± 15	8 ± 4	0.029
AUC_0-210_ TOF (mm×min)	7279 ± 2945	6621 ± 3549	−657 ± 584	0.269
iAUC_0-210_ TOF (mm×min)	−3616 ± 3839	−5939 ± 4280	−2323 ± 605	0.001
Fasted satisfaction (mm)	32 ± 15	32 ± 18	0 ± 3	0.952
AUC_0-210_ satisfaction (mm×min)	12,338 ± 3268	13,309 ± 3487	970 ± 513	0.068
iAUC_0-210_ satisfaction (mm×min)	5658 ± 3619	6751 ± 4323	1093 ± 623	0.089

Fasted, AUC and iAUC VAS–appetite responses at Week 0 and Week 8 are reported as mean ± SD (*n* = 32). The change from Week 0 to Week 8 (∆Week 8) is reported as mean ± SEM, analysed using paired T-test. AUC_0-210_, area under the curve measured over 210 min; iAUC_0-210_, incremental AUC measured over 210 min; TOF, thoughts of food; VAS, visual analogue scale.

**Table 3:** Blood biomarkers at Week 0 and Week 8.

Biomarkers	Week 0	Week 8	∆Week 8	*p*-Value
Glucose and glucoregulatory peptides				
Fasted Glucose (mM)	5.7 ± 0.95	5.2 ± 0.5	−0.5 ± 0.2	0.006
AUC_0-210_ Glucose (mM×min)	1160 ± 237	1124 ± 118	−36 ± 37	0.330
iAUC_0-210_ Glucose (mM×min)	−30 ± 81	30 ± 102	61 ± 20	0.004
Fasted Insulin (pg/mL)	981.2 ± 1006.4	985.7 ± 1137.5	4.5 ± 64.5	0.945
AUC_0-210_ Insulin (pg/mL×min)	387,611 ± 242,789	387,372 ± 221,294	−239 ± 19,712	0.990
iAUC_0-210_Insulin (pg/mL×min)	181,711 ± 125,434	180,575 ± 107,820	−1136 ± 124,721	0.939
Fasted Glucagon (pg/mL)	53.9 ± 32.0	43.9 ± 23.4	−9.9 ± 5.2	0.066
AUC_0-210_ Glucagon (pg/mL×min)	15,877 ± 7385	13,550 ± 5336	−2327 ± 1161	0.054
iAUC_0-210_ Glucagon (pg/mL×min)	4562 ± 4192	4330 ± 3134	−232 ± 864	0.790
Fasted GIP (pg/mL)	58.2 ± 52.8	49.9 ± 31.2	−8.3 ± 9.2	0.375
AUC_0-210_ GIP (pg/mL×min)	50,651 ± 16,790	65,093 ± 19,705	14,442 ± 2688	<0.001
iAUC_0-210_ GIP (pg/mL×min)	38,426 ± 16,778	54,617 ± 17,795	16,191 ± 3155	<0.001
Gastrointestinal peptides				
Fasted GLP-1 (pg/mL)	181.0 ± 89.9	164.1 ± 98.3	−17.0 ± 17.2	0.332
AUC_0-210_ GLP-1 (pg/mL×min)	54,909 ± 21,691	56,045 ± 20,956	1136 ± 3734	0.763
iAUC_0-210_ GLP-1 (pg/mL×min)	16,818 ± 13,534	20,222 ± 13,727	3405 ± 3459	0.333
Fasted PYY (pg/mL)	44.9 ± 40.8	45.7 ± 38.9	0.9 ± 3.6	0.807
AUC_0-210_ PYY (pg/mL×min)	12,750 ± 8158	14,031 ± 7697	1281 ± 699	0.076
iAUC_0-210_ PYY (pg/mL×min)	3374 ± 3071	4428 ± 4349	1054 ± 842	0.220
Branched-chain amino acids				
Fasted Leucine (µM)	121.6 ± 27.8	124.4 ± 29.5	2.7 ± 5.8	0.638
AUC_0-210_ Leucine (µM×min)	38,018 ± 6546	37,007 ± 6665	−1011 ± 1188	0.401
iAUC_0-210_ Leucine (µM×min)	12,472 ± 4106	10,889 ± 3834	−1584 ± 927	0.098
Fasted Isoleucine (µM)	64.3 ± 18.4	68.3 ± 19.4	4.0 ± 3.3	0.228
AUC_0-210_ Isoleucine (µM×min)	21,996 ± 4115	22,095 ± 4449	99 ± 696	0.888
iAUC_0-210_ Isoleucine (µM×min)	8498 ± 2904	7754 ± 2503	−745 ± 591	0.217
Fasted Valine (µM)	239.7 ± 62.9	242.3 ± 55.3	2.6 ± 11.1	0.815
AUC_0-210_ Valine (µM×min)	63,751 ± 12,903	62,849 ± 10,001	−902 ± 1891	0.637
iAUC_0-210_ Valine (µM×min)	13,422 ± 4928	11,970 ± 4494	−1452 ± 1147	0.215
Other essential amino acids				
Fasted Phenylalanine (µM)	56.4 ± 10.9	54.0 ± 6.7	−2.4 ± 2.1	0.268
AUC_0-210_ Phenylalanine (µM×min)	151,82 ± 1759	14,407 ± 986	−775 ± 244	0.003
iAUC_0-210_ Phenylalanine (µM×min)	3340 ± 1556	3062 ± 1220	−278 ± 351	0.435
Fasted Methionine (µM)	27.6 ± 9.1	24.2 ± 5.9	−3.3 ± 1.9	0.083
AUC_0-210_ Methionine (µM×min)	6572 ± 1939	5903 ± 1414	−669 ± 378	0.086
iAUC_0-210_ Methionine (µM×min)	786 ± 846	814 ± 726	28 ± 177	0.876
Fasted Lysine (µM)	83.5 ± 14.2	82.1 ± 15.2	−1.4 ± 3.4	0.687
AUC_0-210_ Lysine (µM×min)	22,271 ± 3451	21,541 ± 3926	−731 ± 840	0.391
iAUC_0-210_ Lysine (µM×min)	786 ± 846	814 ± 726	28 ± 177	0.876
Fasted Histidine (µM)	52.3 ± 12.3	58.6 ± 14.0	−6.2 ± 3.2	0.058
AUC_0-210_ Histidine (µM×min)	11,643 ± 2729	12,449 ± 2524	806 ± 586	0.179
iAUC_0-210_ Histidine (µM×min)	656 ± 2165	153 ± 1712	−503 ± 539	0.359
Fasted Threonine (µM)	118.0 ± 23.3	106.9 ± 27.6	−11.1 ± 4.6	0.022
AUC_0-210_ Threonine (µM×min)	27,652 ± 6083	24,870 ± 6143	−2782 ± 1125	0.019
iAUC_0-210_ Threonine (µM×min)	2881 ± 3185	2420 ± 2683	−461 ± 775	0.556
Fasted Tryptophan (µM)	42.8 ± 10.4	37.8 ± 7.1	−5.0 ± 2.0	0.020
AUC_0-210_ Tryptophan (µM×min)	9861 ± 1696	8899 ± 1074	−962 ± 273	0.001
iAUC_0-210_ Tryptophan (µM×min)	884 ± 1344	970 ± 1238	86 ± 352	0.809
Non-essential amino acids				
Fasted Glycine (µM)	237.1 ± 68.2	249.2 ± 72.8	12.1 ± 8.0	0.139
AUC_0-210_ Glycine (µM×min)	49,349 ± 13,475	51,714 ± 14,718	2365 ± 1819	0.203
iAUC_0-210_ Glycine (µM×min)	−434 ± 5734	−612 ± 5522	−178 ± 1339	0.895
Fasted Aspartic acid (µM)	6.0 ± 4.2	4.7 ± 3.1	−1.3 ± 1.0	0.210
AUC_0-210_ Aspartic acid (µM×min)	1218 ± 368	1029 ± 447	−189 ± 99	0.065
iAUC_0-210_ Aspartic acid (µM×min)	107 ± 474	39 ± 662	−68 ± 175	0.698
Fasted Asparagine (µM)	48.1 ± 8.2	47.5 ± 6.4	−0.6 ± 1.8	0.727
AUC_0-210_ Asparagine (µM×min)	12,610 ± 2299	12,159 ± 2018	−451 ± 397	0.266
iAUC_0-210_ Asparagine (µM×min)	2506 ± 2064	2189 ± 1569	−317 ± 430	0.467
Fasted Glutamic acid (µM)	45.3 ± 20.8	35.2 ± 15.7	−10.1 ± 3.9	0.014
AUC_0-210_ Glutamic acid(µM×min)	9408 ± 4765	7400 ± 2627	−2008 ± 684	0.006
iAUC_0-210_ Glutamic acid (µM×min)	−111 ± 3370	4 ± 2947	115 ± 852	0.893
Fasted Glutamine (µM)	551.5 ± 92.0	540.5 ± 77.9	−11.0 ± 14.7	0.460
AUC_0-210_ Glutamine (µM×min)	122,671 ± 18,948	118,172 ± 16,194	−4499 ± 2660	0.101
iAUC_0-210_ Glutamine (µM×min)	6864 ± 11,086	4668 ± 11,034	−2196 ± 2469	0.381
Fasted Arginine (µM)	72.5 ± 13.2	79.0 ± 19.0	6.4 ± 3.6	0.085
AUC_0-210_ Arginine (µM×min)	20,122 ± 3943	20,601 ± 4232	479 ± 901	0.598
iAUC_0-210_ Arginine (µM×min)	4894 ± 4121	4022 ± 2840	−872 ± 900	0.340
Fasted Alanine (µM)	422.2 ± 95.8	347.6 ± 74.9	−74.5 ± 17.4	<0.001
AUC_0-210_ Alanine (µM×min)	94561 ± 20,182	79,243 ± 14,554	−15,318± 4134	<0.001
iAUC_0-210_ Alanine (µM×min)	5909 ± 10,503	6244 ± 12,105	335 ± 2589	0.898
Fasted Serine (µM)	107.3 ± 29.4	122.0 ± 23.8	14.7 ± 5.0	0.006
AUC_0-210_ Serine (µM×min)	25,181 ± 6155	27,432 ± 5421	2251 ± 973	0.028
iAUC_0-210_ Serine (µM×min)	2647 ± 3699	1812 ± 846	−835 ± 1053	0.006
Fasted Tyrosine (µM)	67.7 ± 13.8	58.5 ± 10.4	−9.2 ± 2.3	<0.001
AUC_0-210_ Tyrosine (µM×min)	17,822 ± 2604	16,303 ± 2234	−1518 ± 423	0.001
iAUC_0-210_ Tyrosine (µM×min)	3613 ± 2154	4026 ± 1579	413 ± 479	0.396
Fasted Proline (µM)	230.7 ± 81.5	182.0 ± 64.7	−48.7 ± 8.6	<0.001
AUC_0-210_ Proline (µM×min)	60,677 ± 16,967	53,784 ± 14,143	−6892 ± 1864	<0.001
iAUC_0-210_ Proline (µM×min)	12,220 ± 7582	15,557 ± 4997	3337 ± 1578	0.043
Non-proteogenic amino acids				
Fasted Hydroxyproline (µM)	13.7 ± 6.7	11.5 ± 3.3	−2.2 ± 1.4	0.115
AUC_0-210_ Hydroxyproline (µM×min)	2714 ± 1203	2341 ± 595	−374 ± 238	0.127
iAUC_0-210_ Hydroxyproline (µM×min)	−161 ± 367	−74 ± 225	87± 83	0.302
Fasted Taurine (µM)	95.7 ± 39.4	100.5 ± 38.2	4.9 ± 10.1	0.632
AUC_0-210_ Taurine (µM×min)	16,472 ± 3967	17,009 ± 3313	536 ± 699	0.449
iAUC_0-210_ Taurine (µM×min)	−4054 ± 7619	−4103 ± 8700	−49 ± 2216	0.983
Fasted Citrulline (µM)	28.9 ± 6.0	26.7 ± 6.9	−2.2 ± 1.2	0.071
AUC_0-210_ Citrulline (µM×min)	6021 ± 1196	5631 ± 1118	−390 ± 167	0.026
iAUC_0-210_ Citrulline (µM×min)	−48 ± 865	14 ± 738	63 ± 194	0.749
Fasted Ornithine (µM)	36.0 ± 16.1	32.9 ± 15.6	−3.1 ± 2.5	0.217
AUC_0-210_ Ornithine (µM×min)	11,389 ± 5152	10,047 ± 3190	−1342 ± 681	0.058
iAUC_0-210_ Ornithine (µM×min)	3831 ± 2815	3142 ± 352	−689 ± 513	0.189

Fasted, AUC and iAUC biomarkers at Week 0 and Week 8 are reported as mean ± SD (*n* = 32). The change from Week 0 to Week 8 (∆Week 8) is reported as mean ± SEM, analysed using paired T-test. AUC_0-210_, area under the curve measured over 210 min; iAUC_0-210_, incremental AUC measured over 210 min; GIP, gastric inhibitory polypeptide; GLP-1, glucagon-like peptide-1; PYY, peptide YY.

**Table 4:** Linear regression analysis of the association between the change in AUC biomarkers and AUC appetite responses after adjusting for covariates.

∆AUC Biomarker	∆AUC Hunger				∆AUC Fullness				∆AUC TOF				∆AUC Satisfaction			
	Estimates	*p*-Value	η_p_^2^	Model R^2^	Estimates	*p*-Value	η_p_^2^	Model R^2^	Estimates	*p*-Value	η_p_^2^	Model R^2^	Estimates	*p*-Value	η_p_^2^	Model R^2^
Model 1																
GLP-1 ^a^	0.074 ± 0.034	0.037	0.15	0.44	−0.104 ± 0.032	0.004	0.28	0.39	0.082 ± 0.036	0.029	0.17	0.28	−0.078 ± 0.030	0.017	0.19	0.31
PYY	0.145 ± 0.151	0.346	0.03	0.34	−0.050 ± 0.164	0.763	0.00	0.15	0.101 ± 0.156	0.523	0.02	0.15	−0.060 ± 0.137	0.666	0.01	0.16
GIP	0.071 ± 0.036	0.059	0.12	0.42	−0.075 ± 0.038	0.057	0.12	0.27	0.033 ± 0.039	0.396	0.03	0.17	−0.013 ± 0.034	0.698	0.01	0.17
Valine	0.164 ± 0.063	0.015	0.20	0.45	−0.142 ± 0.071	0.055	0.13	0.25	0.175 ± 0.064	0.011	0.21	0.33	−0.113 ± 0.060	0.069	0.11	0.23
Glycine ^a^	−0.172 ± 0.066	0.014	0.20	0.51	0.109 ± 0.078	0.172	0.07	0.25	−0.113 ± 0.069	0.114	0.09	0.33	0.107 ± 0.064	0.107	0.09	0.27
Proline	−0.169 ± 0.054	0.004	0.26	0.51	0.123 ± 0.059	0.047	0.13	0.38	−0.178 ± 0.057	0.004	0.26	0.37	0.083 ± 0.054	0.136	0.08	0.25
Model 2																
GLP-1 ^a^	0.096 ± 0.031	0.005	0.28	0.67	−0.132 ± 0.031	<0.001	0.43	0.61	0.100 ± 0.034	0.007	0.27	0.55	−0.111 ± 0.028	0.001	0.39	0.57
PYY	0.150 ± 0.139	0.291	0.04	0.53	−0.043 ± 0.158	0.790	0.00	0.32	0.113 ± 0.140	0.428	0.03	0.41	−0.063 ± 0.129	0.633	0.01	0.36
GIP	0.092 ± 0.034	0.013	0.22	0.63	−0.101 ± 0.039	0.016	0.21	0.45	0.030 ± 0.040	0.455	0.02	0.41	−0.023 ± 0.037	0.530	0.02	0.31
Valine	0.125 ± 0.059	0.046	0.15	0.59	−0.111 ± 0.071	0.134	0.09	0.35	0.129 ± 0.062	0.047	0.15	0.49	−0.086 ± 0.060	0.160	0.08	0.35
Glycine ^a^	−0.284 ± 0.055	<0.001	0.53	0.77	0.193 ± 0.082	0.027	0.19	0.43	−0.197 ± 0.067	0.007	0.26	0.57	0.150 ± 0.070	0.042	0.16	0.43
Proline	−0.155 ± 0.059	0.014	0.22	0.62	0.142 ± 0.068	0.047	0.15	0.46	−0.147 ± 0.062	0.026	0.18	0.50	0.083 ± 0.061	0.186	0.07	0.37
Model 3																
GLP-1 ^a^	0.098 ± 0.029	0.003	0.32	0.67	−0.138 ± 0.028	<0.001	0.50	0.64	0.108 ± 0.031	0.002	0.33	0.56	−0.115 ± 0.023	<0.001	0.51	0.69
PYY	0.118 ± 0.141	0.409	0.03	0.51	−0.041 ± 0.162	0.802	0.00	0.28	0.081 ± 0.146	0.586	0.01	0.34	−0.069 ±0.127	0.593	0.01	0.38
GIP	0.093 ± 0.034	0.012	0.23	0.62	−0.108 ± 0.038	0.009	0.25	0.45	0.046 ± 0.039	0.250	0.05	0.37	−0.049 ± 0.033	0.152	0.08	0.41
Valine	0.128 ± 0.061	0.046	0.15	0.57	−0.129 ± 0.073	0.089	0.11	0.34	0.143 ± 0.065	0.037	0.16	0.46	−0.109 ± 0.057	0.066	0.07	0.43
Glycine ^a^	−0.241 ± 0.055	<0.001	0.45	0.74	0.142 ± 0.081	0.092	0.11	0.36	−0.144 ± 0.067	0.043	0.16	0.50	0.126 ± 0.063	0.055	0.15	0.47
Proline	−0.155 ± 0.052	0.006	0.26	0.63	0.126 ± 0.060	0.045	0.15	0.48	−0.152 ± 0.057	0.013	0.22	0.49	0.084 ± 0.051	0.110	0.10	0.47

Mean estimates (±SEM), *p*-value, and partial *r*^2^ (η_p_^2^) are presented for each biomarker of interest, *n* = 32. Model 1: ∆AUC Appetite = Week 0 AUC Appetite + Week 0 AUC Biomarker + ∆AUC Biomarker; Model 2: ∆AUC Appetite = Age + Baseline BW + ∆BW + Week 0 AUC Appetite + Week 0 AUC Biomarker + ∆AUC Biomarker; Model 3: ∆AUC Appetite = Age + Baseline FFM + ∆FFM + Week 0 AUC Appetite + Week 0 AUC Biomarker + ∆AUC Biomarker. ^a^ *n* = 31, after excluding outlier. TOF, thoughts of food; GLP-1, glucagon-like peptide-1; PYY, peptide YY; GIP, gastric inhibitory polypeptide.

## Data Availability

De-identified data may be shared and made available upon reasonable request to the corresponding author and subject to an approved proposal and data access agreement.

## Supplementary Materials

The following supporting information can be downloaded at: https://www.mdpi.com/article/10.3390/nu15102399/s1, Figure S1: Postprandial VAS ratings. Figure S2: Postprandial concentrations of glucose, GI peptides, and glucoregulatory peptides. Figure S3: Postprandial concentrations of amino acids.
