# Supplementary material for: No Evidence That Circulating GLP-1 or PYY Are Associated with Increased Satiety during Low Energy Diet-Induced Weight Loss: Modelling Biomarkers of Appetite
Source: Nutrients. 2023 May 20;15(10):2399. doi: 10.3390/nu15102399 (PMC10221374; doi:10.3390/nu15102399)
Supplement: Supplementary file 1 [file nutrients-15-02399-s001.zip › nutrients-2278179-supplementary.pdf]

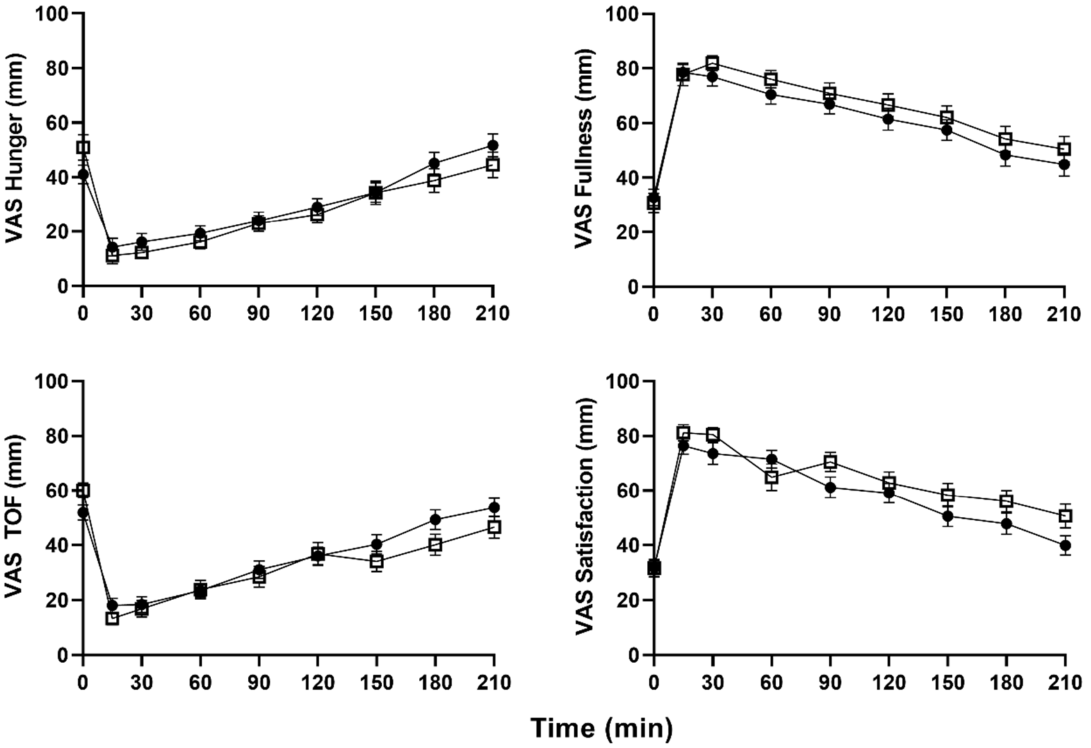

Supplementary Figure S1. Postprandial VAS ratings. Data reported as mean (±SEM) Hunger, Fullness, Thoughts of Food (TOF), and Satisfaction. Close circle represents Week 0; Open square represents Week 8.

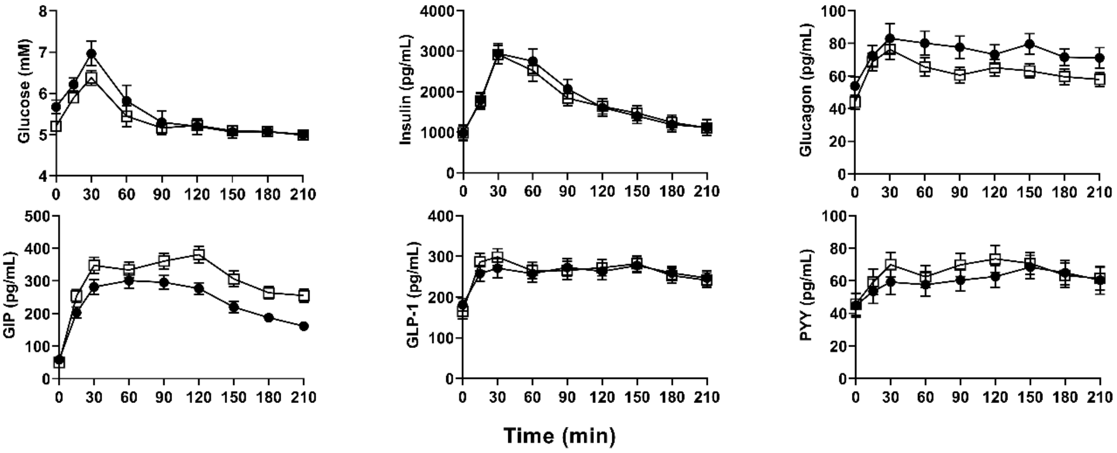

Supplementary Figure S2. Postprandial concentrations of glucose, GI peptides, and glucoregulatory peptides. Data reported as mean (±SEM) Hunger, Fullness, Thoughts of Food (TOF), and Satisfaction. Closed circle represents Week 0; Open square represents Week 8.

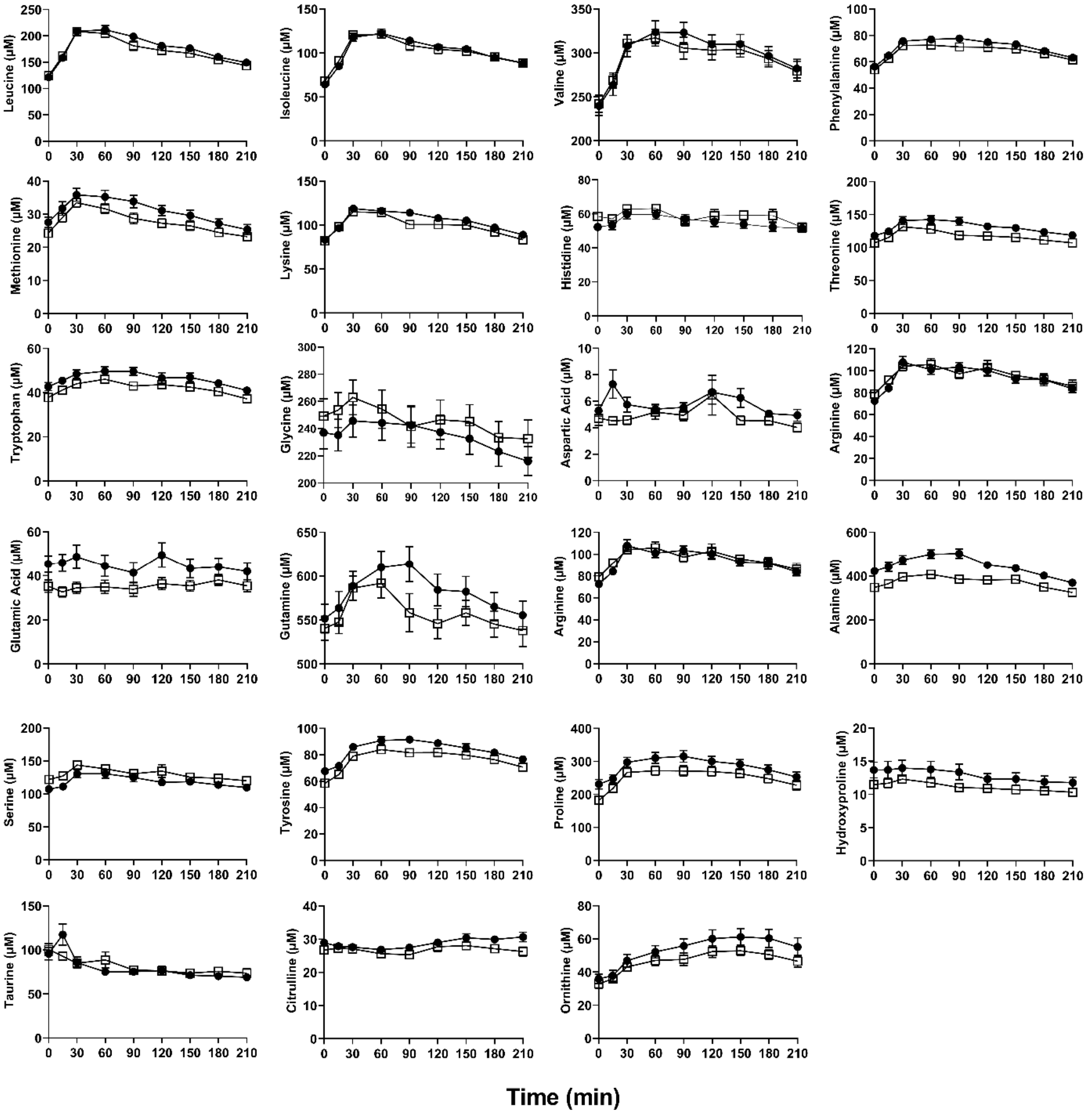

Supplementary Figure S3. Postprandial concentrations of amino acids. Data reported as mean (±SEM). Closed circle represents Week 0; Open square represents Week 8.
